# Supplementary material for: GAK and PRKCD are positive regulators of PRKN-independent mitophagy
Source: Nat Commun. 2021 Oct 20;12:6101. doi: 10.1038/s41467-021-26331-7 (PMC8528926; doi:10.1038/s41467-021-26331-7)
Supplement: Supplementary file 1 — Supplementary Information [file 41467_2021_26331_MOESM1_ESM.pdf]

SUPPLEMENTARY FIGURES

GAK and PRKCD are positive regulators of PRKN-independent mitophagy

Michael J. Munson[1,2,3]*, Benan J. Mathai[1,2,7], Matthew Yoke Wui Ng[1,2,7], Laura Trachsel-Moncho[1,2,8], Laura R. de la Ballina[1,2,8], Sebastian W. Schultz[2,4], Yahyah Aman[5], Alf H. Lystad[1,2], Sakshi Singh[1,2], Sachin Singh[2,6], Jørgen Wesche[2,6], Evandro F. Fang[5] & Anne Simonsen[1,2,4]*

[1]Division of Biochemistry, Department of Molecular Medicine, Institute of Basic Medical Sciences, University of Oslo, N-0372, Oslo, Norway.
[2]Centre for Cancer Cell Reprogramming, Institute of Clinical Medicine, Faculty of Medicine, University of Oslo, N-0316, Oslo, Norway.
[3]Advanced Drug Delivery, Pharmaceutical Sciences, BioPharmaceuticals R&D, AstraZeneca, Gothenburg, Sweden
[4]Department of Molecular Cell Biology, The Norwegian Radium Hospital Montebello, N-0379, Oslo, Norway
[5]Department of Clinical Molecular Biology, University of Oslo and Akershus University Hospital, 1478, Lørenskog, Norway
[6]Department of Tumor Biology, The Norwegian Radium Hospital Montebello, N-0379, Oslo, Norway
[7]These authors contributed equally: Benan J. Mathai, Matthew Yoke Wui Ng
[8]These authors contributed equally: Laura Trachsel-Moncho, Laura R. de la Ballina

*Corresponding Authors:
michael.munson@astrazeneca.com
anne.simonsen@medisin.uio.no

Supplementary Figure 1

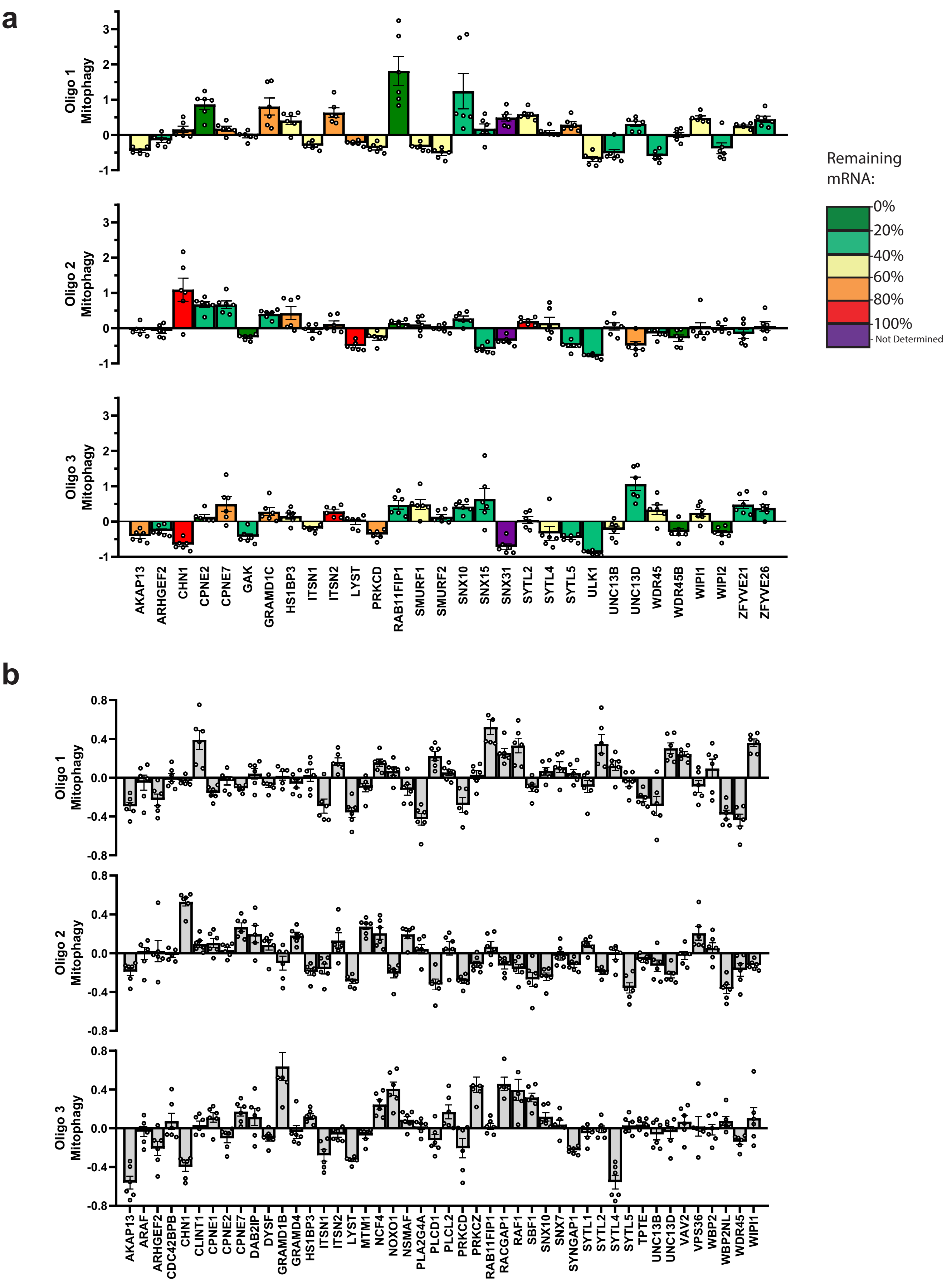

Supplementary Fig. 1 – Secondary and Tertiary siRNA screen

a Secondary screen of targets identified in the primary screen, carried out by transfection of the individual single siRNA oligos (7.5 nM each) for 48 h prior to 24 h 1 mM DFP treatment. Bars represent mean fold change in mitophagy relative to the siNT controls ± SEM from n= 6 plates, colour represents the level of mRNA knockdown ascertained by qPCR analysis where green = high knockdown, red = poor knockdown or purple = not determined. **b** Tertiary siRNA screen carried out by transfection of individual siRNA oligos (15 nM each) for 72 h prior to 24 h 1 mM DFP treatment. Bars represent mean fold change in mitophagy relative to the siNT controls ± SEM from n= 6 plates.

Supplementary Figure 2

a

GAK Interaction Map

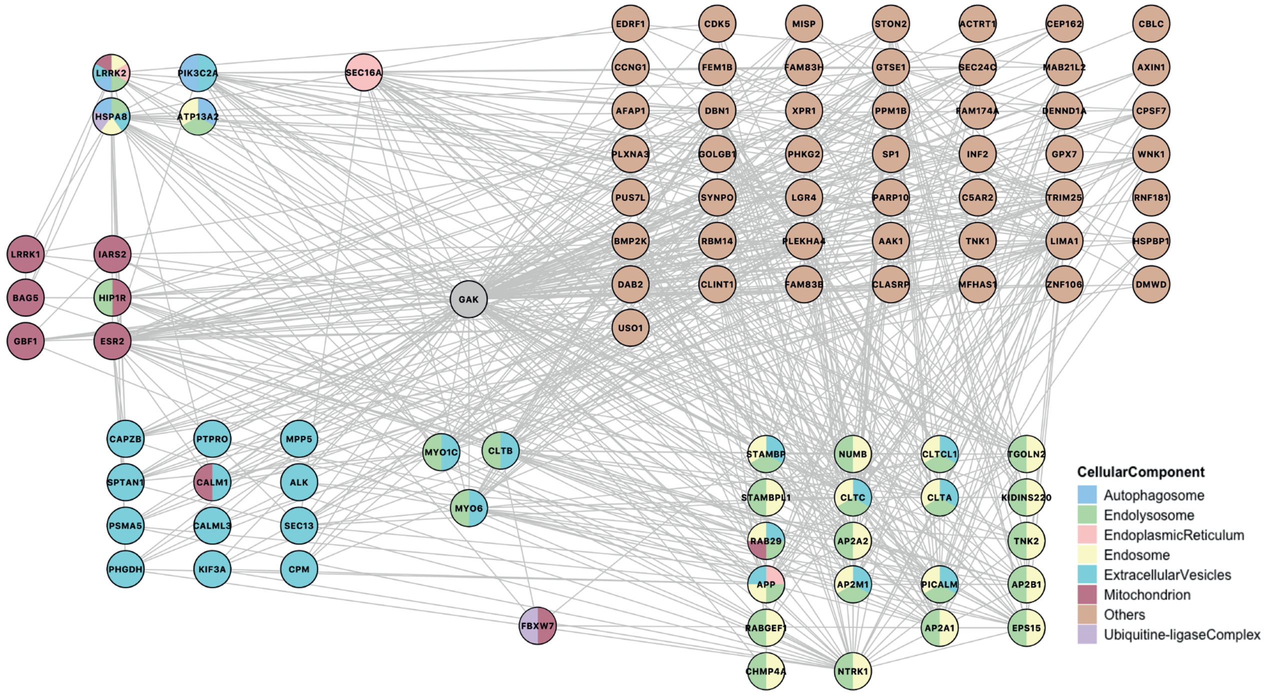

b

PRKCD Interaction Map

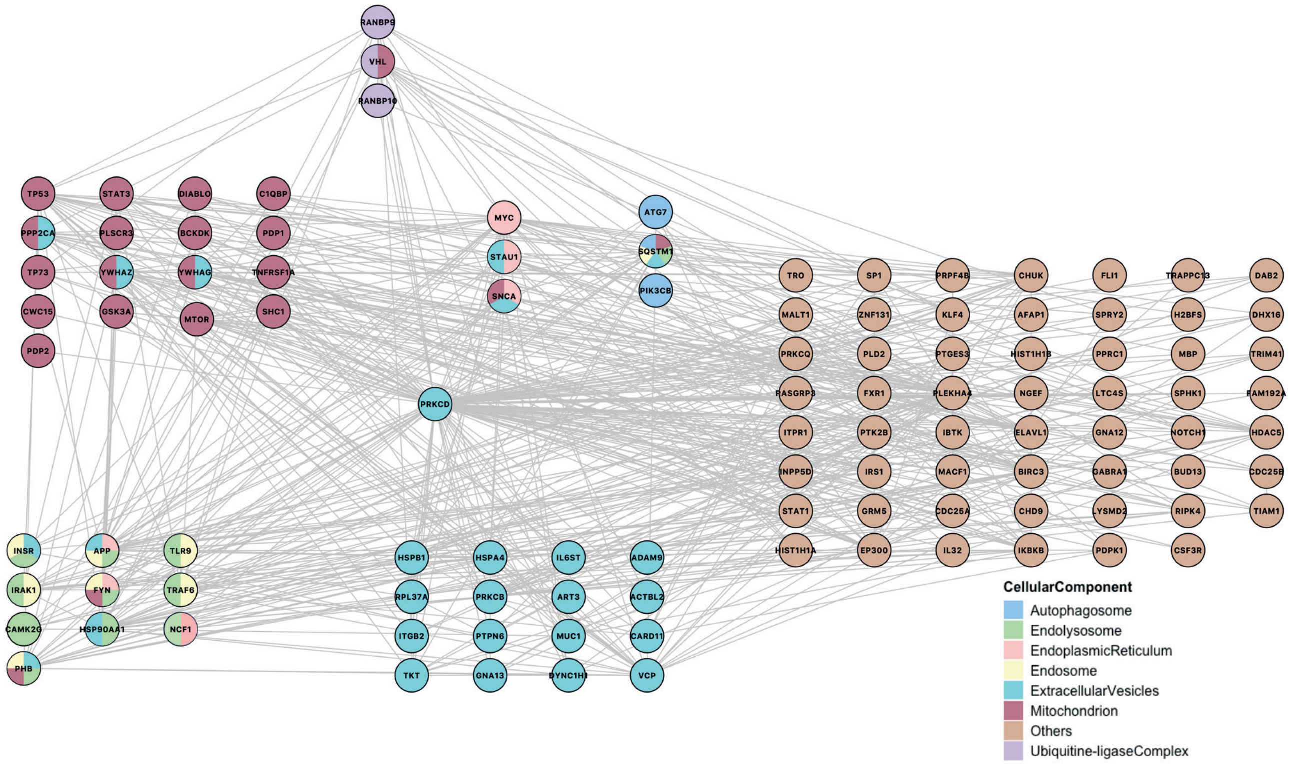

Supplementary Fig. 2 – GAK and PRKCD Protein Interaction Maps

Interaction maps were generated using interaction data obtained from BioGRID (see Methods). Interactors of **a** GAK and **b** PRKCD are shown with GO analysis carried out on interacting proteins to define cellular compartment as shown by grouping and colouring.

Supplementary Figure 3

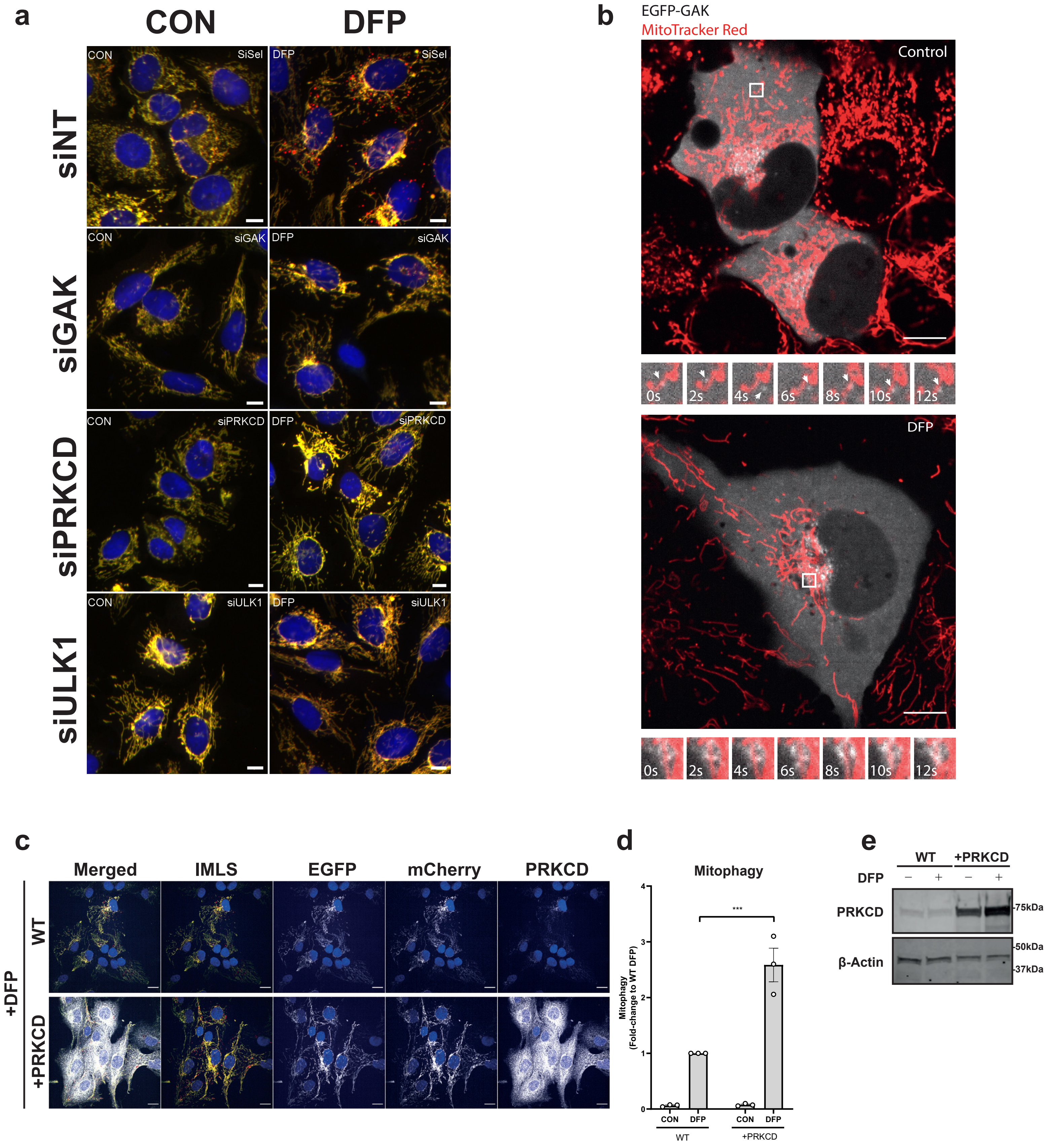

Supplementary Fig. 3 – siRNA screen target examples and exogenous GAK or PRKCD expression

a Representative fluorescence images of siGAK, siPRKCD, siULK1 and siNT control treated U2OS cells ± 1 mM DFP for 24 h and stained with DAPI (blue). Scale bar = 10 µm **b** Transient expression of EGFP-GAK in U2OS cells ± 1 mM DFP for 24 h and co-stained with MitoTracker Red 30 mins prior to live cell imaging with a Dragonfly 505 (Andor) microscope. Scale bar = 10 µm **c** Representative fluorescence images of U2OS IMLS cells (WT) or those overexpressing PRKCD following treatment with 1mM DFP for 24 h and antibody staining for PRKCD. Scale bar = 20 µm. **d** Quantitation of mitophagy from cells as treated in **c.** Data represents mean mitophagy puncta from n=3 independent experiments ± SEM following normalisation to the DFP WT control. Significance was determined by two-way ANOVA followed by Sidak's post-test where *** = p < 0.001. **e** Western blot representing the level of PRKCD overexpression. For precise p-values, see source data file.

Supplementary Figure 4

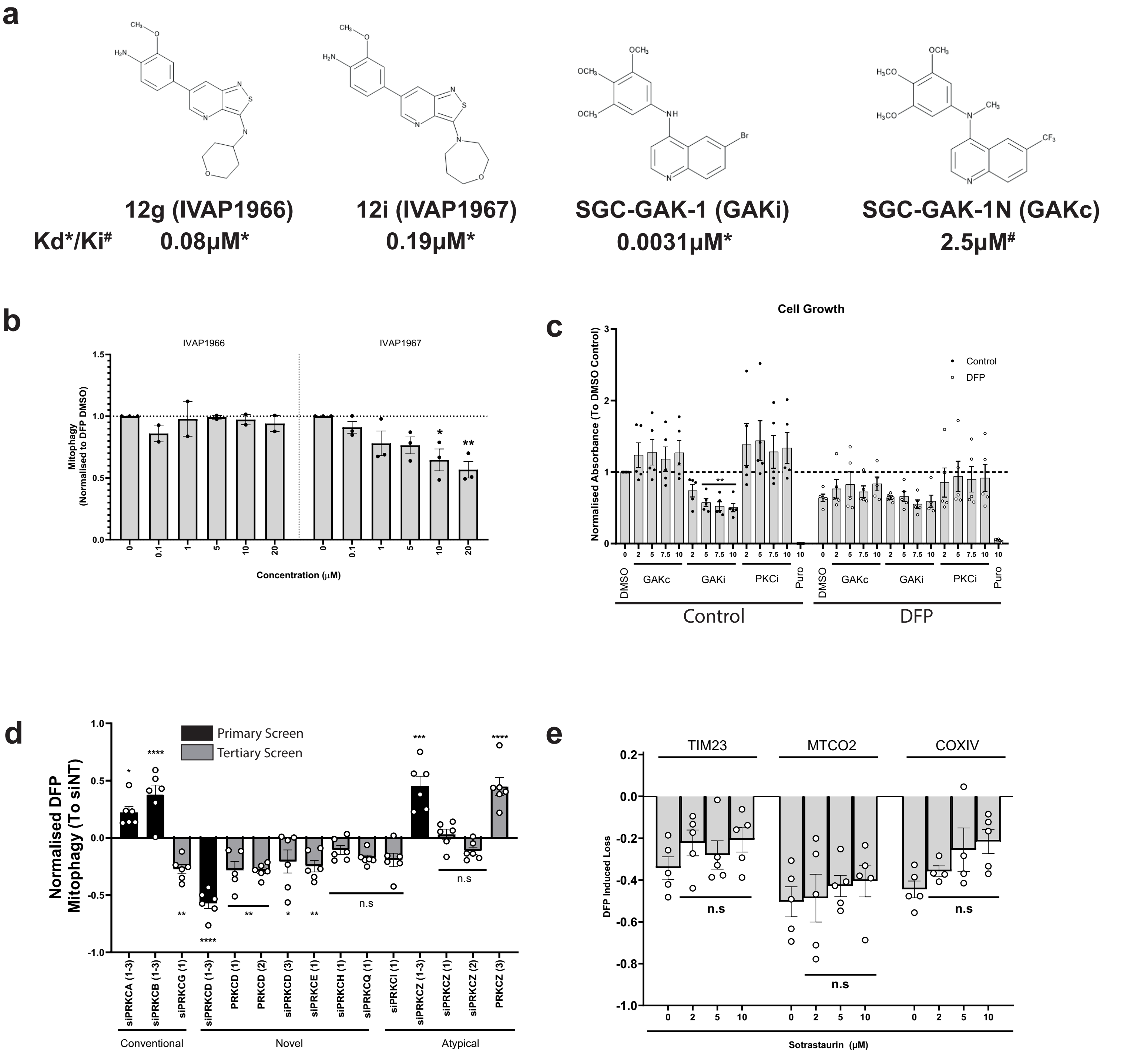

Supplementary Fig. 4 – GAK inhibitors, siPKC and PKCi effect on mitophagy

a Structural comparison of different GAK inhibitors utilised in this study and their Kd/Ki values reported in original publications[29,30]. **b** U2OS IMLS cells were treated for 24h ± 1mM DFP in the presence of indicated kinase inhibitors. Cells were fixed, imaged and quantified for red only structures and plotted relative to DFP DMSO control. Bars represent mean red structures ± SD from n= 2 (IVAP1966) or ± SEM from n=3 (IVAP1967) independent experiments. Significance was determined for IVAP1967 by one-way ANOVA and Dunnett's multiple comparison test to the DMSO control. **c** U2OS cells were treated with indicated concentrations of inhibitors for 24 h prior to crystal violet staining to determine cell viability (see methods). Values represent fold change in cell number relative to DMSO control ± SEM from n=5 independent experiments. **d** U2OS IMLS cells treated with siRNA against indicated PKC isoforms (Primary screen = 7.5 nM, Tertiary = 15 nM) for 48 h prior to induction of mitophagy with 1 mM DFP for 24 h. Value in brackets represents oligonucleotide # used. Values represent mean fold change in mitophagy relative to the siNT control ± SEM from n=6 plates. Significance was determined by one-way ANOVA to the relevant siNT control. **e** U2OS cells were treated as in Fig. 4d ±1mM DFP for 24 h and 0-10 μM Sotrastaurin. Indicated protein abundance was determined relative to loading control and then expressed as a mean DFP-induced loss (DFP-Control values) ± SEM from n=3 independent experiments. Significance was determined by two-way ANOVA followed by Dunnett's post-test to the 0μM control. Where noted, * = $p < 0.05$, ** = $p < 0.01$, *** = $p < 0.001$, **** = $p < 0.0001$ and n.s = not significant. For precise p-values, see source data file.

Supplementary Figure 5

a

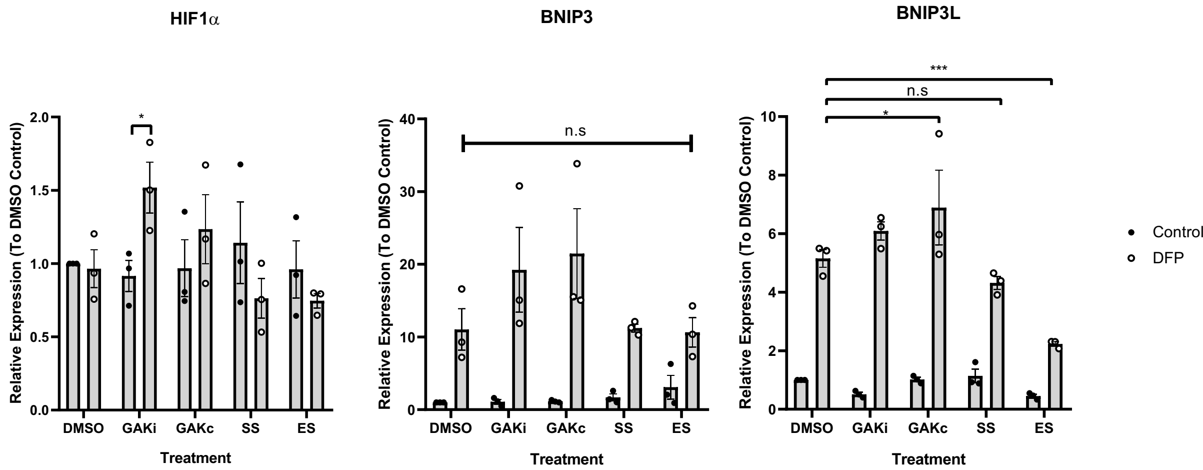

b

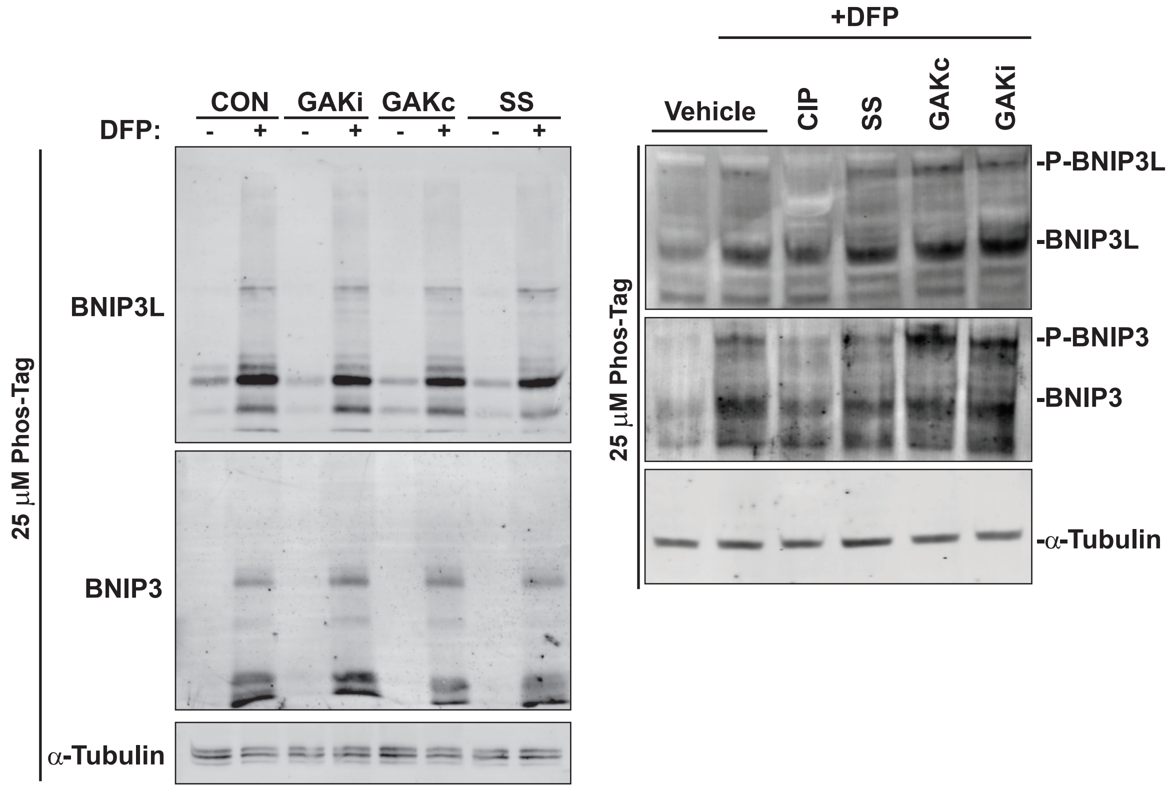

Supplementary Figure 5 - PKCi and GAKi on HIF1α signalling

a U2OS cells were treated ± 1 mM DFP for 24 h in addition to GAKi/GAKc (10 μM), sotrastaurin (SS - 2 μM), enzastaurin (ES - 2μM) or DMSO control prior to RNA isolation. qPCR analysis was carried out to determine the level of BNIP3, BNIP3L and HIF1α transcript levels after normalisation to TATA Binding box protein (TBP) followed by normalisation to the DMSO control. Values represent mean fold change in transcript relative to the DMSO control from n = 3 independent experiments ± SEM. Significance was determined by two-way ANOVA followed by Dunnett's post-test to the DMSO+DFP control (BNIP3/BNIP3L) or Sidak's post-test (HIF1α). **b** U2OS cells were treated ± 1 mM DFP for 24 h with GAKi/GAKc (10 μM each), sotrastaurin (SS - 2 μM) or DMSO control. Samples were ran on an 8 % acrylamide gel containing 25 μM Phos-Tag reagent and blotted for indicated proteins to identify phosphorylation induced band shifts[32]. Where noted, * = $p < 0.05$, *** = $p < 0.001$ and n.s = not significant. For precise p-values, see source data file.

Supplementary Figure 6

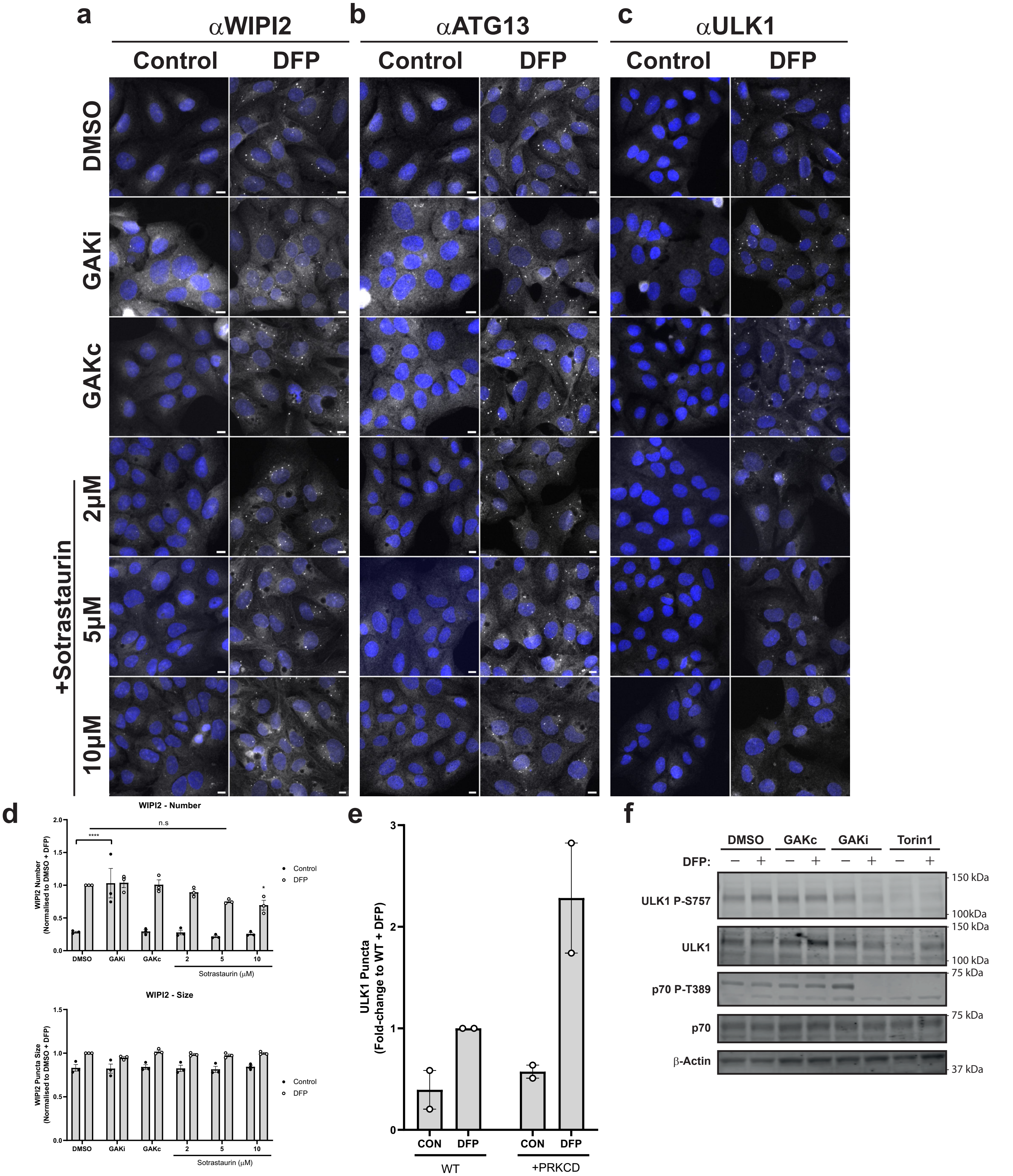

Supplementary Figure 6 – Early autophagic machinery recruitment with GAKi and PKCi treatment

a-c U2OS IMLS cells were treated ± 1mM DFP for 24h ± GAKi (10µM), GAKc (10µM) or Sotrastaurin (2-10µM) prior to PFA fixation and co-staining with antibodies directed against endogenous early autophagy markers **a** WIPI2 **b** ATG13 **c** ULK1. Representative 20x images of cells taken by Zeiss AxioObserver are shown with DAPI staining (blue), scale bar = 10µm. **d** Quantitation of WIPI2 puncta formed in **a** from n=3 independent experiments.Significance was determined by two-way ANOVA followed by Dunnett's post-test to the DMSO control. **e** Quantitation of ULK1 puncta from cells overexpressing PRKCD and treated with 1mM DFP for 24h. Data represents mean ULK1 puncta from n=2 independent experiments ± SD following normalisation to the DFP WT control. **f** U2OS IMLS cells were treated ± 1 mM DFP for 24 h with DMSO, GAKi or GAKc (10µM), or Torin1 (0.25µM). Samples were west-ern blotted for the indicated proteins. Where noted * = p < 0.05, **** = p < 0.0001 and n.s = not significant. For precise p-values, see source data file.

Supplementary Figure 7

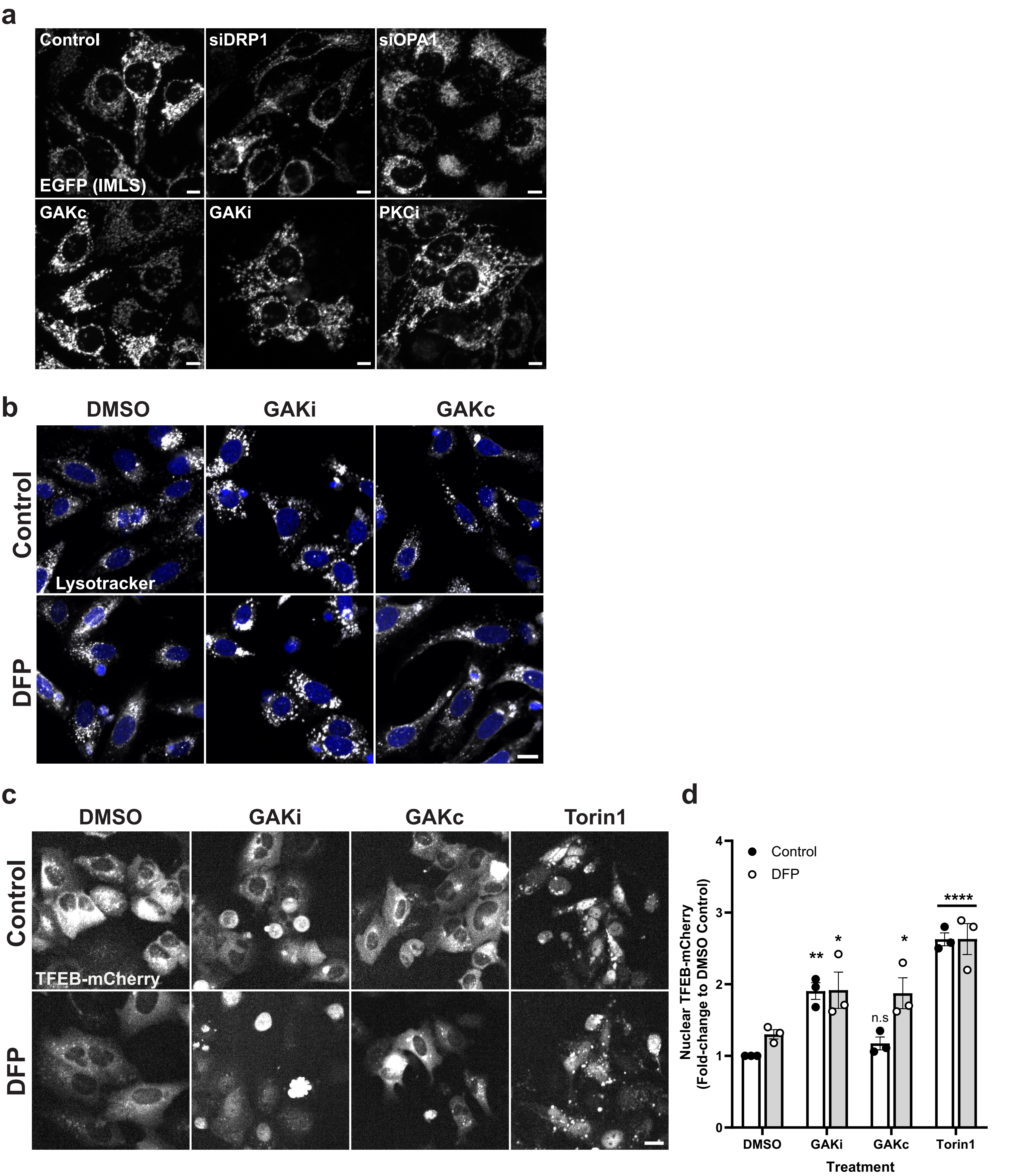

Supplementary Figure 7 – Mitochondrial network classification, TFEB and lysotracker staining

a U2OS IMLS cell mitochondrial network images (EGFP channel) used for training of machine learning classification (Fig. 7b). Cells were treated for 24 h with GAKi (10 µM), GAKc (10 µM) or Sotrastaurin (PKCi – 2µM) compared to 72 h knockdown of non-targeting control, siDRP1 or siOPA1. Scale bar = 10 µm **b** U2OS were treated ± 1mM DFP for 24h with GAKi (10µM), GAKc (10 µM) or DMSO control and 50 nM lysotracker red for the final 1h. Scale bar = 20 µm **c** U2OS cells expressing TFEB-mCherry were treated ± 1mM DFP for 24h with GAKi (10µM), GAKc (10 µM), Torin1 (1µM) or DMSO control. Nuclear TFEB-mCherry was determined by co-staining with Hoechst 33342. Scale bar = 10 µm. **d** Quantitation of cells treated in **c**, mean nuclear TFEB-mCherry relative to the DMSO control treatment ± SEM from n = 3 independent experiments. Significance was determined by two-way ANOVA followed by Dunnett's post-test to the DMSO treatment where * = $p < 0.05$, ** = $p < 0.01$, *** = $p < 0.001$, **** = $p < 0.0001$ and n.s = not significant. For precise p-values, see source data file.

Supplementary Figure 8

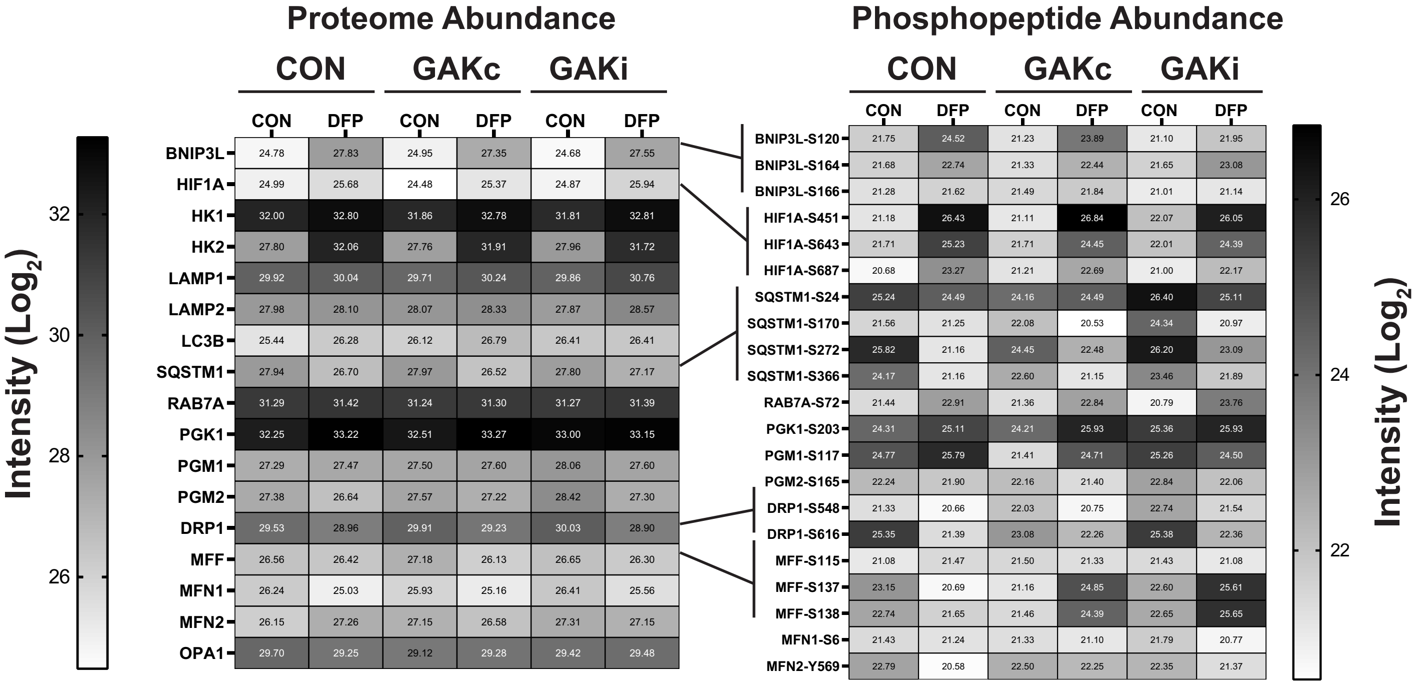

Supplementary Figure 8 - Mass Spectrometry of GAK inhibitors

Mass spectrometry of DFP and GAKi regulated proteins/phospho-peptides. U2OS cells were treated ± 1 mM DFP for 24 h in combination with DMSO vehicle, GAKi or GAKc (both 10 μM). Cell pellets were collected and split between proteome and phospho-peptide analysis (see methods). Data represents average Log_2 intensity from n=4 independent experiments.

Supplementary Figure 9

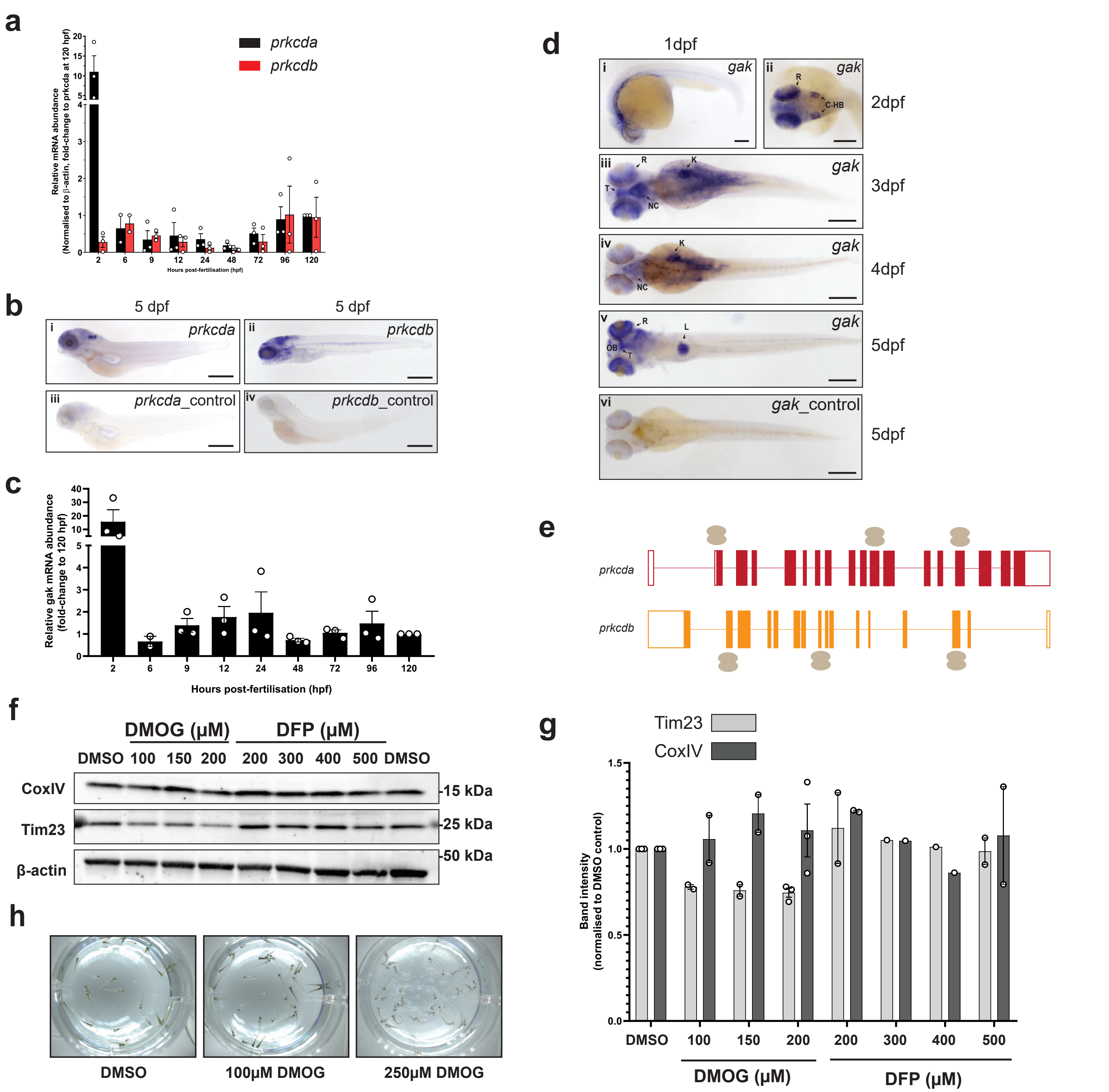

Supplementary Figure 9 – prkcd and mitophagy in zebrafish

a Temporal expression pattern of prkcda and prkcdb. The graph shows the mean relative transcript abundance in whole zebrafish embryos from 2 hpf to 5 dpf ± SD from n=2 (6 hpf) or ± SEM from n=3 (all others) independent experiments **b** Spatial expression pattern of prkcda and prkcdb at 5 dpf as demonstrated by whole mount in situ hybridization (ISH) in lateral view using a 5'UTR targeting probe (i,ii) or negative sense controls (iii,iv). Scale bar = 200 µm. **c** Temporal expression pattern of gak. The graph shows the mean relative transcript abundance in whole zebrafish embryos from 2 hpf to 5 dpf ± SD from n=2 (6 hpf) or ± SEM from n=3 (all others) independent experiments. **d** Spatial expression pattern of gak at 1 dpf to 5 dpf as demonstrated by whole mount ISH at the indicated stages (i-v) using a 3'UTR targeting probe compared to a negative sense control probe (vi). Marked regions indicate retina (R), caudal hindbrain (C-HB), neurocranium (NC), optical tectum (T), kidney (K), olfactory bulb (OB) or liver (L). Scale bar = 200 µm. **e** Crispr-Cas9 manipulations of prkcda and prkcdb gene. Schematic description of the exon (red and orange boxes) and intron (red and orange line) structure of prkcda and prkcdb gene respectively. Double spherical structure with helical strands on specific boxes indicates Cas9 along with sgRNA targeting specific exons. Exon 2, 11 and 15 were targeted to create prkcda KO, whereas exon 2, 8 and 14 were targeted to create prkcdb KO. **f** Representative immunoblots of CoxIV, TIM23 and β-actin on whole embryo lysates of WT larvae treated with varying concentrations of DMOG and DFP for 24 hours at 3 dpf. Control was treated with DMSO for 24 hours. β-actin serves as the loading control. **g** Quantification of the TIM23 and Cox IV signal intensities from blots in **f** normalised to DMSO signal intensity. Bars indicate mean ± SD from n=1 (300, 400µm DFP), n=2 (200, 500µM DFP; 100, 150µM DMOG) or n=3 (DMSO, 200µM DMOG) independent experiments **h** Representative images of control (DMSO) and DMOG treated larvae at 3 dpf.

Supplementary Table 1

Supplementary Table 1 – A selection of mitochondrial proteins and their intensity change (Log_2) between control and DFP treatments from Fig. 1e.

Protein	Localisation	Change (Log_2)
TOMM70A	OMM	-0.1029
TOMM34	OMM	-0.3002
VDAC2	OMM	+0.2473
MRPL16	Matrix	-0.3513
MRPL27	Matrix	-1.5596
COQ9	Matrix	-0.5090
TIMM23	IMM	-1.4452
TIMM8B	IMM	-0.6625
NDUFA12	IMM	-1.4433

Supplementary Table 2

Supplementary Table 2 – Oligonucleotide primers and probes utilised in this study.

Target	Usage	Forward (5' -> 3')	Reverse (5' -> 3')
AKAP13	qPCR	GCAGAGCCCAGAATGTGAGA	CCATGTCATCACTGGGTGAGT
ARHGEF2	qPCR	TTCTCAGGTCCTAGTGCGGA	CGGGTCACTTTCCGGATGAA
β-actin	ZF, qPCR	CGAACGACCAACCTAAACCTCTCG	ATGCGCCATACAGAGCAGAAGC
BNIP3	qPCR	GGCCATCGGATTGGGGATCT	GGCCACCCCAGGATCTAACA
BNIP3L	qPCR	TCCACCCAAGGAGTTCCACT	GTGTGCTCAGTCGCTTTCCA
CHN1	qPCR	CTAAAGAGAGTGACCCTCCACG	GGGTGGGTCCAAAGACGATT
CPNE2	qPCR	GTGTGCTCAGTCGCTTTCCA	AGGATGAAGTACTGCGTGGC
CPNE7	qPCR	AGGATGAAGTACTGCGTGGC	AGGCCTCTGTGGCTGTAGTA
GAK	qPCR	GTGGAGGAAGAGATCACGAGG	AGATATCCTGCTTCTCGCCG
GRAMD1C	qPCR	GCAACTGCTCCAGCAGAACTA	CTTCTTCCTGGACTTCTTGGCT
HIF1α	qPCR	CTTCTTCCTGGACTTCTTGGCT	GCAGGGTCAGCACTACTTCG
HS1BP3	qPCR	Qiagen – QT00094899	
ITSN1	qPCR	CGGAGATGAGGCGTCGATTA	ACTGCTGATCATGCTTCGCT
ITSN2	qPCR	GGCTCAGTTTCCCACAGCTA	ACGTGCTTGATCACCTGTTATGT
LYST	qPCR	Qiagen – QT00094906	
PRKCD	qPCR	GCAGGGTCAGCACTACTTCG	GCAGGGTCAGCACTACTTCG
prkcda	ZF, qPCR	TTGGCGTATCTGTGTTGGCT	ATGACGACACACTGAGCCTG
prkcdb	ZF, qPCR	ACAAAAAATGTGCCAGCGGAC	ACGCGAACTGTGGAGACAAT
prkcda	5'UTR ISH Probe	GCTCTGCCTGAGGGGTTGCCATGGC	GAGGCCTTGTTTGACAAGACCC
prkcda	ORF ISH Probe	CTGCTGGCAGAAGCTCTTACTCAAG	GAAGGTTGTGGCCCGATTGTCTCC
prkcda	sgRNA #1 (Exon 2)	taatacgactcactataGGGGCCATGGCAACCCCTCgttttagagctagaa	
prkcda	sgRNA #2 (Exon 11)	taatacgactcactataGGGTGGGTGATTCGAGATGGgttttagagctagaa	
prkcda	sgRNA #3 (Exon 15)	taatacgactcactataGGGAGTTCCGCAGAAGGTTGgttttagagctagaa	
prkcdb	5'UTR ISH Probe	GAAGCTGTGATCTCTCACCATG	CCGCAGAGGTTGGCAACCTTTG
prkcdb	ORF ISH Probe	CGGAGGGCTCTCAGTATGGG	GGACGTCCATGCGGATGGAG
prkcdb	sgRNA #1 (Exon 2)	taatacgactcactataGGTCACCATGGCTCCGTTCCTGgttttagagctagaa	
prkcdb	sgRNA #2 (Exon 8)	taatacgactcactataGGTAGCCTGGTACGCGGTTTgttttagagctagaa	
prkcdb	sgRNA #3 (Exon 14)	taatacgactcactataGGGCCTCTTTGGTGATCCAGgttttagagctagaa	
RAB11FIP1	qPCR	GCAGGGTCAGCACTACTTCG	GCAGGGTCAGCACTACTTCG
SMURF1	qPCR	TACCGGATACCAGCGTTTGG	ACCACTATCTGGCCACGAAC
SMURF2	qPCR	CATGTCTAACCCCGGAGGC	CAAATGGATCAGGAAGTCGGAAAA
SYTL2	qPCR	CTGTCCATTTGGCATCGGGA	GTGCTGTCTTCCGCTTCAGA
SYTL4	qPCR	ATGTGCCTGGAACTGACTGT	ACTGATCCCAGTGCCAACAC
SYTL5	qPCR	GTGACAAAATCGCGCAGCTA	GGACAACATCAGTGCCGAGA
SNX10	qPCR	CAGAAAGTTCATCCTCTGGGCT	CAGAAAGTTCATCCTCTGGGCT
SNX15	qPCR	GATGACTTCCTGCGGCACTA	ACCACCACCTCTTTGACATCC
TBP	qPCR	CAGAAAGTTCATCCTCTGGGCT	TATATTCGGCGTTTCGGGCA
UNC13B	qPCR	CTCTGCGTGCGCGTTAAAAG	GGAAGGCTGATCACCACGAA
UNC13D	qPCR	CTCAAGGCAGAACAGCAGGA	GAAGACAGAGCGGAACCTCC
WIPI1	qPCR	CCATAAGGCTGAACCGGCA	CATAGACCTGTTGGGTTTGCAG
WIPI2	qPCR	TGCTCGCTAGCCACAATTCA	TGCTTCATCAGGGCACACTC
WDR45B	qPCR	GCCGAAATACCCTCCCAACA	CCACAATTCTATCTCGCCGC
WDR45	qPCR	TCTGTCATTGCCATCTGCGT	CACGTCGAAAGCCTCTCTGT
zFYVE21	qPCR	GTCATGTCCTCCGAGGTGTC	CATCTCCGACACTCCTTGTCC
zFYVE26	qPCR	CCTCAGAGGGAGAAACGATCAG	TACTGGAGATACCAGGAGGAGC
