# Supplementary material for: GAK and PRKCD are positive regulators of PRKN-independent mitophagy
Source: Nat Commun. 2021 Oct 20;12:6101. doi: 10.1038/s41467-021-26331-7 (PMC8528926; doi:10.1038/s41467-021-26331-7)
Supplement: Supplementary file 3 — Description of Additional Supplementary Files [file 41467_2021_26331_MOESM3_ESM.pdf]

Description of additional supplementary information:

Title; Supplementary Data 1

Description: Raw data and information from primary siRNA screen shown in Fig. 2. Table demonstrates gene name, reference sequence, contained lipid binding domains, and siRNA oligonucleotide sequences that were pooled for targeting each protein.

Title; Supplementary Movie 1

Description: U2OS IMLS cells were treated ± 1mM DFP for 24h in combination with DMSO, GAKi (10µM) or GAKc (10µM). Cells were imaged by widefield across 0-10min as indicated in timestamp. Scale bar = 10µm.
