# Supplementary material for: GAK and PRKCD are positive regulators of PRKN-independent mitophagy
Source: Nat Commun. 2021 Oct 20;12:6101. doi: 10.1038/s41467-021-26331-7 (PMC8528926; doi:10.1038/s41467-021-26331-7)
Supplement: Supplementary file 6 — Reporting Summary [file 41467_2021_26331_MOESM6_ESM.pdf]

nature research

Corresponding author(s): Michael Munson
Anne Simonsen

Last updated by author(s): Sep 1, 2021

Reporting Summary

Nature Research wishes to improve the reproducibility of the work that we publish. This form provides structure for consistency and transparency in reporting. For further information on Nature Research policies, see our Editorial Policies and the Editorial Policy Checklist.

Statistics

For all statistical analyses, confirm that the following items are present in the figure legend, table legend, main text, or Methods section.

n/a	Confirmed	
☐	☒	The exact sample size (*n*) for each experimental group/condition, given as a discrete number and unit of measurement
☐	☒	A statement on whether measurements were taken from distinct samples or whether the same sample was measured repeatedly
☐	☒	The statistical test(s) used AND whether they are one- or two-sided<br>*Only common tests should be described solely by name; describe more complex techniques in the Methods section.*
☒	☐	A description of all covariates tested
☒	☐	A description of any assumptions or corrections, such as tests of normality and adjustment for multiple comparisons
☐	☒	A full description of the statistical parameters including central tendency (e.g. means) or other basic estimates (e.g. regression coefficient) AND variation (e.g. standard deviation) or associated estimates of uncertainty (e.g. confidence intervals)
☐	☒	For null hypothesis testing, the test statistic (e.g. *F*, *t*, *r*) with confidence intervals, effect sizes, degrees of freedom and *P* value noted<br>*Give P values as exact values whenever suitable.*
☒	☐	For Bayesian analysis, information on the choice of priors and Markov chain Monte Carlo settings
☒	☐	For hierarchical and complex designs, identification of the appropriate level for tests and full reporting of outcomes
☒	☐	Estimates of effect sizes (e.g. Cohen's *d*, Pearson's *r*), indicating how they were calculated

Our web collection on statistics for biologists contains articles on many of the points above.

Software and code

Policy information about availability of computer code

Data collection	ZEN Blue (v2.3, Zeiss)<br>ZEN Black (2012 SP5 FP3, Zeiss)<br>IMOD (v4.9) - CLEM<br>FLUOstar OPTIMA (v2.20R2)<br>Image Lab Touch (v2.0.0.27, Bio-Rad)
Data analysis	CellProfiler (v2.8.0, The Broad Institute)<br>CellProfiler Analyst (2.2.1, The Broad Institute)<br>MaxQuant (v1.6.1.0)<br>Perseus (v1.6.5.0)<br>Prism (v9.0.0, Graphpad)<br>Cytoscape (v3.8.0)<br>FIJI (1.52n)<br>FigureJ (v1.10b)<br>Image Studio (v5.2, Bio-Rad)<br>ShinyGO (v0.66)

For manuscripts utilizing custom algorithms or software that are central to the research but not yet described in published literature, software must be made available to editors and reviewers. We strongly encourage code deposition in a community repository (e.g. GitHub). See the Nature Research guidelines for submitting code & software for further information.

Data

Policy information about availability of data

All manuscripts must include a data availability statement. This statement should provide the following information, where applicable:

- Accession codes, unique identifiers, or web links for publicly available datasets
- A list of figures that have associated raw data
- A description of any restrictions on data availability

Data supporting the findings of this study are available within the paper and supplementary information files. Raw data for Fig. 1b,d,f; Fig.2a-f; Fig.3a-d,f-h; Fig.4b-f; Fig.5b-d,f,g; Fig.6e-g; Fig.7b,d,g ; Fig.8c; Fig.9e,g,h; Fig.S1a,b; Fig.S3d,e; Fig.S4b-e; Fig.S5a,b; Fig.S6d-f; Fig.S7d; Fig.S8a; Fig.S9a,c,f,g is available in the source data file.
The proteomics data generated in this study have been deposited in the PRIDE database under accession code PXD022773 (http://proteomecentral.proteomexchange.org/cgi/GetDataset?ID=PXD022773).
ExPASY PROSITE was used to identify proteins containing specific domain sequences using the following identifiers: C1 domains (ID: PDOC00379, https://prosite.expasy.org/PDOC00379), C2 domains (ID: PDOC00380, https://prosite.expasy.org/PDOC00380), ENTH (ID: PDOC50942, https://prosite.expasy.org/PDOC50942), PH Domain (ID: PDOC50003, https://prosite.expasy.org/PDOC50003), PX Domain (ID: PDOC50195, https://prosite.expasy.org/PDOC50195), FYVE domain (PDOC50178, https://prosite.expasy.org/PDOC50178).

Field-specific reporting

Please select the one below that is the best fit for your research. If you are not sure, read the appropriate sections before making your selection.

☒ Life sciences ☐ Behavioural & social sciences ☐ Ecological, evolutionary & environmental sciences

For a reference copy of the document with all sections, see nature.com/documents/nr-reporting-summary-flat.pdf

Life sciences study design

All studies must disclose on these points even when the disclosure is negative.

Sample size	No formal sample-size calculation was performed. Cell biology experiments were generally performed in at least 3x independent biological replicates (for precise details, see figure legends and "Statistics and reproducibility" section of methods). The number of animals used for C.elegans/Zebrafish experiments are noted in figure legends and are consistent with current practices.
Data exclusions	During microscopy imaging experiments, some field of view level data points were omitted due to microscopy autofocus errors/acquisition errors.
Replication	The reported results were replicable, the precise number of experimental repeats are detailed in the figure legends and "Statistics and reproducibility" section
Randomization	During siRNA screening, technical replicates were distributed across the plate in different locations to minimise potential well-plate effects. For all other experiments, cells/animals were randomly assigned to experimental groups. Studies of wild-type vs prkcd_ab DKO zebrafish were defined by injection with control guide or guides targeting prkcd_ab (animals randomly assigned to each group and subsequent treatment groups).
Blinding	No blinding was applied as quantitation for cell experiments and animal experiments was performed utilising automated image analysis tools (CellProfiler). This ensured the same criteria for object and intensity detection was being applied equally across experimental samples and negated bias. Other experiments were not blinded as the results were not subjective.

Reporting for specific materials, systems and methods

We require information from authors about some types of materials, experimental systems and methods used in many studies. Here, indicate whether each material, system or method listed is relevant to your study. If you are not sure if a list item applies to your research, read the appropriate section before selecting a response.

Materials & experimental systems

n/a	Involved in the study	
☐	☒	Antibodies
☐	☒	Eukaryotic cell lines
☒	☐	Palaeontology and archaeology
☐	☒	Animals and other organisms
☒	☐	Human research participants
☒	☐	Clinical data
☒	☐	Dual use research of concern

Methods

n/a	Involved in the study	
☒	☐	ChIP-seq
☒	☐	Flow cytometry
☒	☐	MRI-based neuroimaging

Antibodies

Antibodies used

Primary Antibodies:
ATG13 (Cell Signaling Technology, #13468, Clone E1Y9V)
AMPK P-T172 (Cell Signaling Technology, #2535, Polyclonal)
β-Actin (Cell Signaling Technology, #3700, Clone 8H10D10)
BNIP3 (Cell Signaling Technology, #44060, Clone D7U1T)
BNIP3L (Cell Signaling Technology, #12396, Clone D4R4B)
COXIV (Cell Signaling Technology, #4850, Clone 3E11)
p70 (Cell Signaling Technology, #9202, Polyclonal)
p70 P-T389 (Cell Signaling Technology, #9205, Polyclonal)
PDH (Cell Signaling Technology, #2784, Polyclonal)
PRKCD (Cell Signaling Technology, #9616, D10E2, Polyclonal)
PRKCD (Abcam, #Ab182126, Clone EPR17075)
PRKCD P-S663 (Cell Signaling Technology, #9376, Polyclonal)
LC3B (Cell Signaling Technology, Western blotting only, #3868, Clone D11),
ULK1 (Cell Signaling Technology, #8054, Polyclonal)
ULK1 P-S555 (Cell Signaling Technology, #5869, Polyclonal)
ULK1 P-S757 (Cell Signaling Technology, #6888, Clone D1H4)
FUNDC1 (Abcam, #Ab74834), Polyclonal)
GAK (Abcam, #Ab115179, Clone 1C2)
NIPSNAP1 (Abcam, #Ab67302, Polyclonal)
MTCO2 (Abcam, #Ab110258, Clone 12C4F12)
WIPI2 (Abcam, #Ab105459, Clone 2A2)
ATG13 P-S318 (Rockland, #600-401-C49, Polyclonal)
TIM23 (BD Biosciences, 611223, Clone 32)
LAMP1 (Santa Cruz Biotechnology, sc-20011, Clone H4A3)
TOM20 (Santa Cruz Biotechnology, sc-17764, Clone F-10)
α-Tubulin (Sigma Aldrich, T5168, Clone B-5-1-2)
Chondroitin Sulphate (Abcam, Ab11570, Clone CS-56)
LC3B (MBL International, PM036, Polyclonal)

Secondary Antibodies:

Starbright Blue Anti-Mouse (Bio-Rad, 12004158)
Starbright Blue Anti-Rabbit (Bio-Rad, 12004161)
Rhodamine a-tubulin (Bio-Rad, 12004166)
DyLight 800, Anti-Rabbit (ThermoFisher Scientific, SA5-10044)
DyLight 680, Anti-Rabbit (ThermoFisher Scientific, SA5-10042)
DyLight 680, Anti-Mouse (ThermoFisher Scientific, SA5-10170)
Alexa Fluor-594, Anti-Rabbit (Invitrogen, A11058)
Alexa Fluor-647, Anti-Rabbit (ThermoFisher Scientific, A21245)
Alexa Fluor-647, Anti-Mouse (ThermoFisher Scientific, A21236)

Validation

ATG13 (Cell Signaling Technology, #13468, Clone E1Y9V) - 24x Product Citations on manufacturer's website
AMPK P-T172 (Cell Signaling Technology, #2535, Polyclonal) - 1597x Product Citations on manufacturer's website
β-Actin (Cell Signaling Technology, #3700, Clone 8H10D10) - 1445x Product Citations on manufacturer's website
BNIP3 (Cell Signaling Technology, #44060, Clone D7U1T) - 16x Product Citations on manufacturer's website
BNIP3L (Cell Signaling Technology, #12396, Clone D4R4B) - 28x Product Citations on manufacturer's website
COXIV (Cell Signaling Technology, #4850, Clone 3E11) - 224x Product Citations on manufacturer's website
p70 (Cell Signaling Technology, #9202, Polyclonal) - 1245x Product Citations on manufacturer's website
p70 P-T389 (Cell Signaling Technology, #9205, Polyclonal) - 1177x Product Citations on manufacturer's website
PDH (Cell Signaling Technology, #2784, Polyclonal) - 35x Product Citations on manufacturer's website
PRKCD (Cell Signaling Technology, #9616, D10E2, Polyclonal) - 24x Product Citations on manufacturer's website, KO validated, no cross-reaction with other PKC isoforms.
PRKCD (Abcam, #Ab182126, Clone EPR17075) - 24x product references, KO validated.
PRKCD P-S663 (Cell Signaling Technology, #9376, Polyclonal) - 38x Product Citations on manufacturer's website
LC3B (Cell Signaling Technology, Western blotting only, #3868, Clone D11) - 746x Product Citations on manufacturer's website
ULK1 (Cell Signaling Technology, #8054, Polyclonal) - 264x Product Citations on manufacturer's website
ULK1 P-S555 (Cell Signaling Technology, #5869, Polyclonal) - 179x Product Citations on manufacturer's website
ULK1 P-S757 (Cell Signaling Technology, #6888, Clone D1H4) - 186x Product Citations on manufacturer's website
FUNDC1 (Abcam, #Ab74834), Polyclonal) - 4x Product Citations on manufacturer's website
GAK (Abcam, #Ab115179, Clone 1C2) - Validation in multiple cell lines
NIPSNAP1 (Abcam, #Ab67302, Polyclonal) - 1x Product Citation, validates with brain tissue lysate
MTCO2 (Abcam, #Ab110258, Clone 12C4F12) - 158x Product Citations on manufacturer's website
WIPI2 (Abcam, #Ab105459, Clone 2A2) - 28x Product Citations on manufacturer's website
ATG13 P-S318 (Rockland, #600-401-C49, Polyclonal) - 11x Product Citations on manufacturer's website
TIM23 (BD Biosciences, 611223, Clone 32) - 25x Product Citations
LAMP1 (Santa Cruz Biotechnology, sc-20011, Clone H4A3) - 289x Product Citations on manufacturer's website
TOM20 (Santa Cruz Biotechnology, sc-17764, Clone F-10) - 413x Product Citations on manufacturer's website
α-Tubulin (Sigma Aldrich, T5168, Clone B-5-1-2) - 2470x Product Citations on manufacturer's website
Chondroitin Sulphate (Abcam, Ab11570, Clone CS-56) - 46x product references on manufacturer's website
LC3B (MBL International, PM036, Polyclonal) - 113x Product references on manufacturer's website

Eukaryotic cell lines

Policy information about cell lines

Cell line source(s)	U2OS FlpIn Cells were generated and obtained from Prof. Stephen Blacklow and were used as a basis for derivative cell lines (IMLS -/+ PRKN expression). HEK293FT cells were from Invitrogen. U2OS cells used for generation of TFEB-mCherry cells were from ATCC.
Authentication	Cell Lines were not authenticated
Mycoplasma contamination	Cells tested negative for mycoplasma
Commonly misidentified lines (See ICLAC register)	No commonly misidentified lines were used

Animals and other organisms

Policy information about studies involving animals; ARRIVE guidelines recommended for reporting animal research

Laboratory animals	Wild-type zebrafish (AB strain) and transgenic tandem-tagged mitofish (TT-mitofish) were utilised in accordance with the Norwegian regulation on animal experimentation. All experiments on zebrafish were carried out at or prior to 5 dpf.<br>C.elegans strain N2 Bristol were used. Animals were hermaphrodite and tested at day 2.
Wild animals	Study did not involve wild animals
Field-collected samples	Study did not involve field-collected samples
Ethics oversight	No ethical approval is required for work with c.elegans.<br>As all zebrafish experimentation was carried out at or prior to 5 dpf, no ethical approval was required.

Note that full information on the approval of the study protocol must also be provided in the manuscript.
